# Rivaroxaban versus dalteparin for the treatment of cancer-associated venous thromboembolism: a systematic review and meta-analysis

**DOI:** 10.1097/MS9.0000000000003008

**Published:** 2025-02-07

**Authors:** Bibek Shrestha, Suzit Bhusal, Grishma Kandel, Sudip Bastakoti, Krishna Kumar Yadav

**Affiliations:** a Maharajgunj Medical Campus, Institute of Medicine, Tribhuvan University, Kathmandu, Nepal; b Department of Critical Care Medicine, Mayo Clinic, Jacksonville, Florida, USA; c Teaching Hospital, Tribhuwan University, Kathmandu, Nepal

**Keywords:** anticoagulants, cancer, dalteparin, deep vein thrombosis, rivaroxaban, venous thromboembolism

## Abstract

**Background and aims:**

Traditionally, low molecular weight heparin, such as dalteparin, has been the first-line treatment for cancer-associated venous thromboembolism (VTE). However, recent studies suggest that rivaroxaban, a direct oral anticoagulant, may offer comparable efficacy with the convenience of oral administration. This systematic review and meta-analysis aim to evaluate and compare the efficacy and safety of rivaroxaban versus dalteparin in managing cancer-associated VTE, focusing on recurrence rates, bleeding events, and patient adherence.

**Methods:**

This review follows the Preferred Reporting Items for Systematic Reviews and Meta-Analysis Protocols guidelines, systematically retrieving data from PubMed and Cochrane databases using structured search terms related to cancer, VTE, and anticoagulation. Data on recurrent VTE, major bleeding, and patient outcomes were extracted. Statistical analyses were performed using a random-effects model to account for heterogeneity.

**Results:**

Nine studies were analyzed, encompassing a range of study designs across multiple countries. The findings show no significant difference in major bleeding risk between rivaroxaban and dalteparin (risk ratio: 0.91, *P* = 0.69), nor in bleeding-related mortality (odds ratio [OR]: 4.00, *P* = 0.36). Rivaroxaban was associated with a significant reduction in deep vein thrombosis (DVT) recurrence (OR: 0.75, *P* = 0.04) and a marginally nonsignificant reduction in pulmonary embolism recurrence (OR: 0.73, *P* = 0.05). Nonsignificant bleeding events were slightly higher with rivaroxaban but did not reach statistical significance.

**Conclusion:**

Rivaroxaban presents a viable alternative to dalteparin for treating cancer-associated VTE, showing comparable safety regarding major bleeding and potential efficacy in reducing DVT recurrence. This study supports the potential for more standardized guidelines that include rivaroxaban as a feasible option in cancer-associated VTE management.

## Introduction

Venous thromboembolism (VTE) is a severe and fatal condition encompassing deep vein thrombosis (DVT) and pulmonary embolism (PE). It is defined as the formation of clots in the venous system with incidences increased in the population with malignancy, immobilization, surgical intervention, and chemotherapy.^[1,2]^ Patients with active cancers are more vulnerable to developing VTE, with studies suggesting that metastatic conditions can even double the risk of VTE if compared to those without cancer.^[3]^ Pathophysiology in increasing the risk of DVT in malignant conditions involves a combination of tumor-related factors, tumor-related characteristics, and patient-related characteristics that increase the likelihood of thrombus formation.^[4]^ Cancer cells can also directly activate blood coagulation by producing procoagulant factors, which include releasing inflammatory cytokines and interacting with host vascular cells.^[5]^ The activation of this homeostatic pathway can not only increase the thrombosis risk but also promote tumor growth. The severity of VTE in cancer patients is also profound as both the condition DVT and PE can even lead to significant mortality as well as morbidity. Cancer patients with VTE exhibit a higher 1-year case fatality rate of 63.4% compared to 12.6% in non-cancer patients.^[6]^ VTE includes immediate as well as long-term complications, which include post-thrombotic syndrome, which can severely affect the quality of life. Therefore, effective management of VTE in cancer patients is vital as it can enhance the quality of life and reduce the mortality rates which are related to thromboembolic complications.^[7]^

**Highlights**

- Rivaroxaban was associated with a statistically significant reduction in the recurrence of deep vein thrombosis (DVT) compared to dalteparin, suggesting it may be more effective in preventing recurrent DVT events among cancer patients.
- The study found no significant difference in major bleeding risks between rivaroxaban and dalteparin, indicating that rivaroxaban may provide a safe alternative to dalteparin with respect to major bleeding events.
- Rivaroxaban, being an oral direct anticoagulant, offers a more convenient alternative to dalteparin, which requires injection. This convenience could enhance patient adherence to anticoagulant therapy, especially for long-term treatment in cancer patients.

Anticoagulation therapy is one of the crucial cornerstones of VTE management in cancer patients; with low molecular weights heparin (LMWH), such as dalteparin, is traditionally the preferred treatment option due to its efficacy and lower risk of bleeding as compared to oral anticoagulants. The standard guideline also recommends LMWH as the first-line treatment for VTE in cancer patients.^[8]^ Recently, direct oral anticoagulants (DOACs), particularly rivaroxaban, have emerged as an efficient alternative for managing cancer-related VTE. Rivaroxaban also has advantages, such as the oral drug administration route, which increases the patient’s convenience and adherence to medication. The interest in DOACs, especially rivaroxaban, has surged due to recent clinical trials indicating its effectiveness in treating cancer-associated VTE. Research has shown that rivaroxaban may offer efficacy that is at least comparable to that of LMWHs, with the added benefit of once-daily oral dosing, which can significantly improve patient adherence and overall quality of life.^[9]^ The convenience of oral administration, combined with a favorable pharmacokinetic profile, positions rivaroxaban as an attractive option for managing VTE in cancer patients, particularly those who may struggle with the logistics of injectable therapies.^[10]^ Despite the promising data surrounding DOACs, there remains an ongoing debate regarding the optimal choice between rivaroxaban and dalteparin for cancer-associated VTE. Clinical guidelines exhibit variability, and there is a lack of consensus on the best treatment approach for this high-risk population.^[8]^ Variations in clinical practice are evident, with some practitioners favoring LMWHs due to their established safety and efficacy, while others advocate for DOACs based on emerging evidence.^[11]^ This uncertainty is also reflected in the variation in the developed clinical guidelines, which lack the preferred and recommended anticoagulant in the high-risk population. Given these challenges, a systematic review and meta-analysis of the existing literature comparing rivaroxaban and dalteparin for cancer-associated VTE is warranted. This study aims to provide a comprehensive synthesis of current evidence, focusing on both efficacy and safety outcomes, to support more informed decision-making in clinical practice. By evaluating pooled data across various studies, this review seeks to clarify whether rivaroxaban can be a viable and potentially preferable alternative to dalteparin in treating VTE in cancer patients. Ultimately, this study could contribute to developing more standardized treatment guidelines and improving the quality of care for cancer patients experiencing VTE.

## Methods

This systematic review has been conducted following the Preferred Reporting Items for Systematic Reviews and Meta-Analysis Protocols which is provided in the supplemental digital content.^[12]^. The Risk of Bias in Systematic Reviews and Assessment of multiple systematic reviews AMSTAR 2 were followed when doing this meta-analysis.^[13]^ Since the article type was systematic review and meta-analysis, institutional review board approval was not necessary. The study was registered in research registry with unique identifying number approved.

### Data source and strategy

The search through the electronic database was conducted in June 2024. Two major electronic databases, including PubMed and Cochrane Library, were used for the searching by two independent authors. Updated and comprehensive search strategy was developed by using the key search terms and synonyms. In PubMed, Keywords and Terms included four domains of population, intervention, comparator, and outcome. Population included the following mesh term: Cancer, Cancer-associated, and Oncology patients whereas Intervention included: Rivaroxaban and Comparator included Dalteparin and Outcome included Venous Thromboembolism (VTE), Deep Vein Thrombosis (DVT), Pulmonary Embolism (PE). The search strategy used was (“Venous thromboembolism” OR “VTE” OR “Pulmonary Embolism” OR “Deep Vein Thrombosis” OR “Blood Clots”) AND (“Cancer-associated” OR “Malignancy” OR “Cancer patients” OR “Oncologic” OR “Neoplasm”) AND (“Direct oral anticoagulant” OR “DOAC” OR “Novel oral anticoagulant” OR “NOAC” OR “Rivaroxaban” OR “Apixaban” OR “Edoxaban” OR “Dabigatran”) AND (“Low molecular weight heparin” OR “LMWH” OR “Heparin” OR “Dalteparin” OR “Enoxaparin” OR “Tinzaparin”).

### Study selection

Inclusion criteria focused on studies involving adults (≥18 years) with cancer-associated VTE, treated with rivaroxaban or dalteparin, comparing their safety and efficacy. Both randomized controlled trials (RCTs) and observational studies were considered, provided they offered direct comparisons of these anticoagulants. Key outcomes included the incidence of recurrent VTE, major bleeding events, and secondary outcomes like survival rates, treatment adherence, and quality of life. Studies excluding cancer diagnosis, involving non-cancer-associated VTE, or lacking direct comparisons between rivaroxaban and dalteparin were omitted to ensure relevant, targeted findings for this high-risk population. This selection aimed to capture evidence for optimal anticoagulation strategies in cancer patients requiring complex care (Table 1).

**Table 1:** Inclusion and exclusion criteria table for our study

PICOS Element	Inclusion criteria	Exclusion criteria	Rationale
Population (P)	Adults (≥18 years) are diagnosed with cancer-associated VTE, including deep vein thrombosis (DVT) and/or pulmonary embolism (PE).	Patients without a confirmed cancer diagnosis.	Cancer patients are at high risk for VTE due to cancer-related hypercoagulability. This population has unique anticoagulation needs and elevated bleeding risk, requiring targeted analysis for optimal anticoagulant selection.
		Pediatric patients or adults with non-cancer-associated VTE.	
	Cancer patients undergoing treatment or in remission.	Studies focusing exclusively on non-VTE thromboembolic events.	
Intervention (I)	Rivaroxaban as the primary intervention for VTE treatment.	Studies using anticoagulants other than rivaroxaban as the main intervention.	Rivaroxaban, a DOAC, is oral, possibly improving adherence. Understanding its efficacy and safety in cancer-associated VTE compared to traditional options like dalteparin is essential for evidence-based recommendations.
	Studies in which rivaroxaban is administered as monotherapy or combined with other cancer therapies.	Studies focused solely on other direct oral anticoagulants (DOACs) without including rivaroxaban.	
Comparator (C)	Dalteparin as the comparator for VTE treatment.	Studies lacking a direct comparison to dalteparin.	Dalteparin is a well-studied LMWH for cancer-associated VTE, often preferred for its safety profile in this population. Including dalteparin allows a direct comparison to the oral option, rivaroxaban.
	Studies include other low-molecular-weight heparins (LMWHs) only if dalteparin is explicitly included or represented.	Comparisons involving DOACs alone without LMWHs.	
Outcomes (O)	Primary outcomes:	Studies without data on primary outcomes (recurrent VTE or major bleeding).	Evaluating recurrent VTE and major bleeding provides insights into the treatment’s safety and effectiveness. Secondary outcomes like survival, adherence, and quality of life give a comprehensive view of the treatment impact, especially important in cancer patients needing complex care.
	• Incidence of recurrent VTE		
	• Incidence of major bleeding events		
	Secondary outcomes:	Outcomes not relevant to anticoagulant safety or efficacy, such as unrelated biomarkers or nonclinical outcomes.	
	• Overall survival rate		
	• Patient adherence to treatment		
	• Quality of life measures		
	• Incidence of minor bleeding events		
Study Design (S)	Randomized controlled trials (RCTs) and observational studies (cohort, case-control) comparing rivaroxaban and dalteparin in cancer-associated VTE patients.	Studies without direct comparison of rivaroxaban and dalteparin.	Including RCTs and observational studies allows for a well-rounded analysis of the evidence. RCTs offer high-quality data, while observational studies provide real-world insights, which is essential for assessing practical treatment impact.
		Case reports, case series, or studies lacking a control group.	
		Nonhuman studies or animal studies.	

### Data extraction and assessment of study quality

Two independent reviewers (B.S. and G.K.) conducted data extraction from the selected studies in Microsoft Excel 365, capturing essential study characteristics such as study design, publication date, journal, country, study duration, cancer type, mean participant age with standard deviation (SD), gender distribution (male to female ratio), and total sample size. Additionally, key outcome events, including major bleeding, bleeding-related mortality, recurrent VTE, recurrent pulmonary embolism (PE), and nonsignificant bleeding, were systematically extracted. The quality assessment of the studies was performed across five domains using the Cochrane Risk of Bias Tool. The risk of bias (ROB) evaluation in this systematic review is central to establishing the reliability of the findings, as it rigorously assesses the methodological integrity of the included studies and gauges the potential influence of bias on the reported outcomes.

### Statistical analysis

RevMan (version 5.3; Copenhagen: Nordic Cochrane Centre, The Cochrane Collaboration) was used for all statistical calculations. We pooled ORs with 95% confidence interval (CI) with Mantel–Haenszel random-effects weighted methods. Random-effects model in our meta-analysis is driven by our commitment to provide a more cautious and comprehensive synthesis of the data. It acknowledges and accommodates the expected heterogeneity among the included studies, thereby yielding a more conservative and robust estimate of the overall treatment effect. We assessed heterogeneity across studies by using Higgins *I*^2^.

### Quality assessment of the included studies

Each study was evaluated by two independent reviewers (B.S. and S.B.) using tailored criteria suited to the study design, following the Cochrane Risk of Bias Tool for the RCTs and Newcastle–Ottawa Scale for observational and retrospective studies.^[14,15]^ For the RCTs, observational study and cohort study, domains assessed included D1 (bias arising due to randomization), D2 (bias due to deviation from intended intervention), D3 (bias due to missing outcome data), D4 (bias in the measurement of the outcome), D5 (bias in the selection of the reported result), overall (overall ROB).

## Results

**Figure 1.**
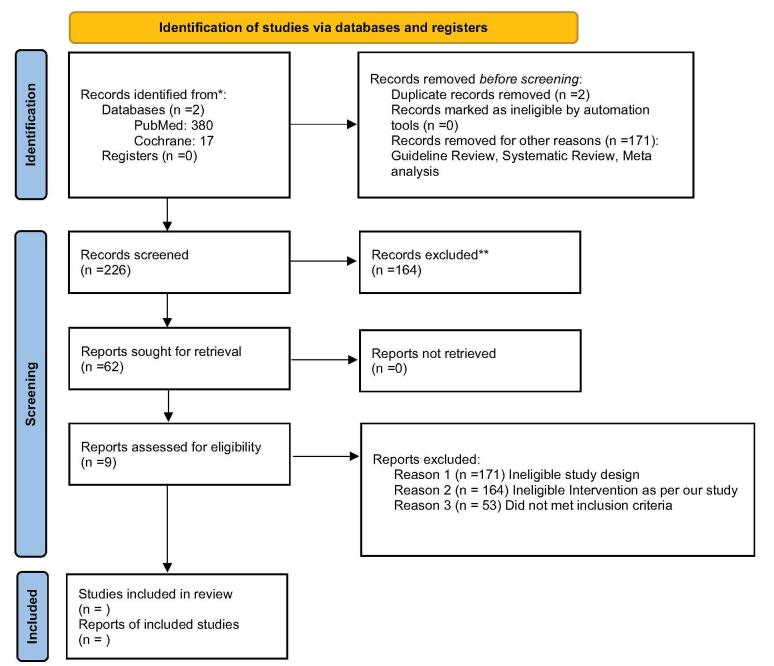
PRISMA 2020 flow diagram of our study.

A total of 397 records were identified from two databases, PubMed (380) and Cochrane^[16]^. Before screening, two duplicate records were removed, along with 171 records excluded for other reasons, including guideline reviews, systematic reviews, and meta-analyses. This left 226 records for title and abstract screening, where 164 were excluded based on initial eligibility criteria. Sixty-two reports were sought for retrieval, all of which were successfully retrieved for full-text assessment. After the full-text screening, records were excluded due to ineligible study design (n = 171), ineligible intervention (n = 164), or failing to meet inclusion criteria (n = 53). Ultimately, nine studies were included in the final review and thoroughly analyzed (Fig. 1).

### Detailed description of the studies included

**Figure 2.**
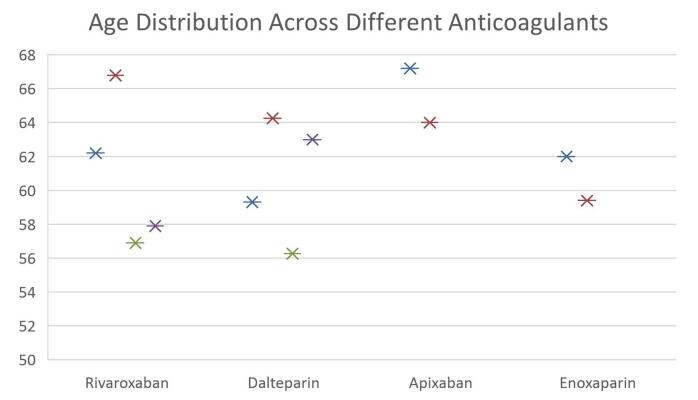
Age distribution.

**Figure 3.**
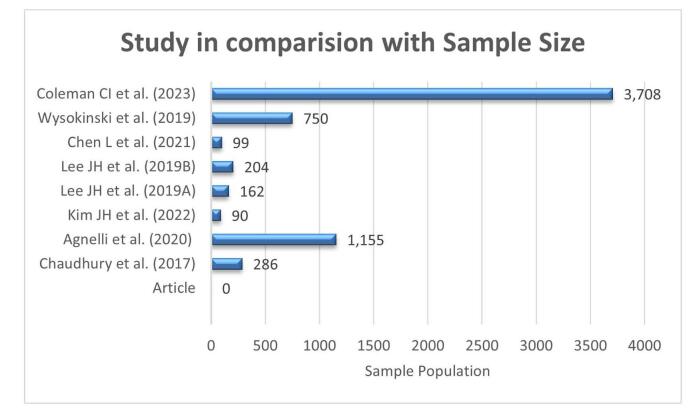
Study in comparision with sample population.

This review included the studies from multiple countries which include four studies conducted in the United States, three studies in South Korea, and one each spanning multiple European countries and Israel, and one in China. The dataset includes four retrospective studies, two observational studies, two randomized, open-label studies, and one prospective, multicenter, randomized study. This diverse design mix enhances both practical insights and controlled findings. The age distribution among anticoagulants, and comparison according to sample size have been shown in Figs. 2 and 3. Key variables such as date of publication, journal, country, study duration, cancer type, participants’ mean age (with SD), male-to-female ratio, and total sample size were systematically extracted during the review process and are summarized inTable 2.

**Table 2:** Characteristics of included study

Article	Study design	Date of publication	Journal	Country	Study duration	Cancer type	Age (mean with SD) of participants	Male: female	Total sample
Chaudhury *et al* (2017)^[17]^	Retrospective, single-center, study	October 27, 2017	*Indian Journal of Hematology and Blood Transfusion*	United States	May 1, 2010–June 30, 2015	Mixed (various types, with a significant proportion having metastatic disease)	Rivaroxaban group: 62.20 ± 12.34 years; dalteparin group: 59.31 ± 12.02 years	Rivaroxaban group: 52.3% male; dalteparin group: 50.8% male	286 patients
Agnelli *et al* (2020)^[18]^	Multinational, randomized, open-label	April 23, 2020	*The New England Journal of Medicine*	Nine European countries, Israel, and the United States	April 2017–June 2019	Mixed types, including advanced and metastatic cancers; exclusion of primary brain tumors, intracerebral metastases, and acute leukemia	Apixaban group: 67.2 ± 11.3 years; dalteparin group: 67.2 ± 10.9 years	Apixaban group: 50.7% male; dalteparin group: 47.7% male	1155 patients
Kim *et al* (2022)^[19]^	Prospective, multicenter, randomized, open-label	January 22, 2022	*Cancers*	South Korea	August 2017–June 2020	Advanced upper gastrointestinal, hepatobiliary, and pancreatic cancers	DOAC group: median age 64 years; dalteparin group: median age 63 years	DOAC group: 56.8% male; dalteparin group: 50% male	90 patients
Lee *et al* (2019a)^[20]^	Retrospective, single-center study	January 2020	*Journal of Gynecologic Oncology*	South Korea	January 1, 2012–December 31, 2017	Primary gynecologic cancers (including ovarian, cervical, uterine, vaginal, vulvar, endometrial, and fallopian tube cancers)	Rivaroxaban group: 56.90 ± 12.32 years; dalteparin group: 56.27 ± 10.34 years	Not applicable as the study focused on gynecologic cancers	162 patients
Lee *et al* (2019b)^[21]^	Retrospective, single-center study	May 16, 2019	*Respiration*	South Korea	January 1, 2012–December 31, 2016	Primary lung cancer	Rivaroxaban group: 66.79 ± 11.32 years; dalteparin group: 64.25 ± 8.66 years	N/A	204 patients
Chen *et al* (2021)^[16]^	Observational study	March 2021	*Journal of the College of Physicians and Surgeons Pakistan*	China	January 2017–September 2019	Mixed (includes gastric, esophageal, gynecologic, lung, colorectal, breast, cholangiopancreatic, and prostate cancers)	67 years (range 34–84 years)	45.5% male and 54.5% female	99 patients
Wysokinski *et al* (2019)^[22]^	Observational study	2019	*American Journal of Hematology*	United States	March 2013–January 2018	Mixed (includes gastrointestinal, pancreatic, genitourinary, hematologic, lung, and other cancers)	Rivaroxaban group: 62 ± 13 years; Enoxaparin group: 62 ± 12 years	Rivaroxaban group: 49.7% female; enoxaparin group: 42.4% female	750 patients
Coleman *et al*. (2023)^[23]^	Retrospective Study	April 2023	*JACC: CardioOncology*	United States	January 2012–December 2020	Mixed (excluded high-bleeding-risk cancers [e.g. esophageal, gastric, bladder])	N/A	N/A	3708 patients
Nicklaus *et al* (2018)^[24]^	Retrospective, single-center	2018	*Journal of Oncology Pharmacy Practice*	United States	January 2012–August 2015	Mixed (primarily solid tumors, with 8% hematological malignancies)	Rivaroxaban group: 57.9 years; enoxaparin group: 59.4 years	Rivaroxaban group: 42% male, 58% female	90 patients

### Major bleeding

**Figure 4.**
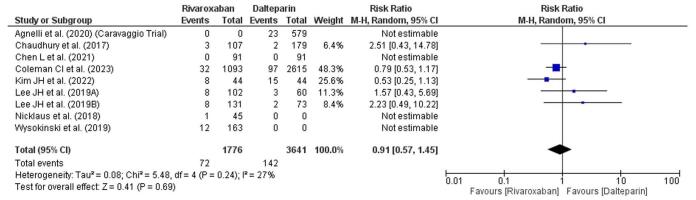
Forest plot showing major bleeding.

The forest plot compares the risk of major bleeding events between rivaroxaban and dalteparin across multiple studies (Fig. 4). The overall risk ratio (RR) is 0.91 (95% CI: 0.57–1.45), indicating no significant difference in major bleeding risk between the two treatments. Heterogeneity among studies is low (*I*^2^ = 27%), suggesting consistency in the results. Individual study estimates vary, with almost 95% CIs crossing the line of no effect (RR = 1), showing nonsignificant results in either direction. The pooled effect, also nonsignificant (*P* = 0.69), does not favor either treatment regarding major bleeding risk.

### Bleeding related mortality

**Figure 5.**
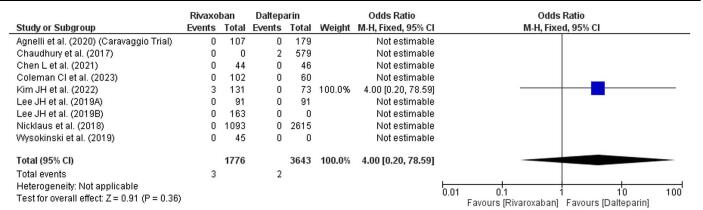
Forest plot showing bleeding-related mortality.

Bleeding-related mortality was evaluated between rivaroxaban and dalteparin across studies, with only a few events reported in each treatment group. (Fig. 5) The overall odds ratio (OR) is 4.00 (95% CI: 0.20–78.59), showing a high degree of uncertainty due to wide CIs, reflecting limited data and low event rates. The heterogeneity measure is not applicable here as only a few studies contributed estimable data, and all CIs span the null value (OR = 1), indicating non-significance. The pooled effect test (*P* = 0.36) is also nonsignificant, suggesting no definitive difference in bleeding-related mortality between the treatments.

### Nonsignificant bleeding event

**Figure 6.**
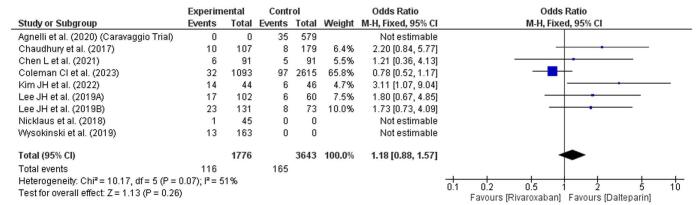
Forest plot showing nonsignificant bleeding.

This forest plot assesses nonsignificant bleeding events between rivaroxaban and dalteparin (Fig. 6). The overall OR is 1.18 (95% CI: 0.88–1.57), indicating a slightly higher, yet statistically nonsignificant, likelihood of nonsignificant bleeding events in the rivaroxaban group compared to dalteparin. The CIs of individual studies overlap with the null effect (OR = 1), suggesting no clear advantage for either treatment in terms of reducing nonsignificant bleeding. The heterogeneity measure is moderate (*I*^2^ = 51%), implying variability among studies but not enough to negate pooled findings. The test for the overall effect is nonsignificant (*P* = 0.26), supporting the conclusion that there is no substantial difference in the risk of nonsignificant bleeding between the two treatments.

### Recurrence of DVT

**Figure 7.**
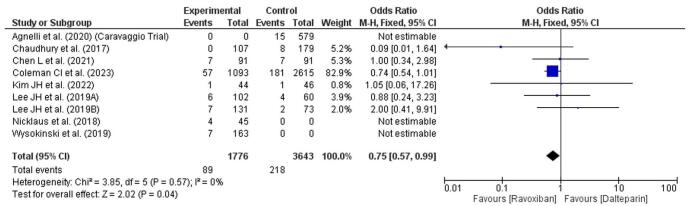
Forest plot showing recurrence of DVT.

Recurrence of DVT was evaluated between patients treated with rivaroxaban and dalteparin (Fig. 7). The overall OR is 0.75 (95% CI: 0.57–0.99), suggesting a statistically significant 25% reduction in the odds of DVT recurrence in the rivaroxaban group compared to the dalteparin group. The CIs barely exclude 1, indicating a borderline significant result (*P* = 0.04). Heterogeneity is minimal (*I*^2^ = 0%), indicating consistent findings across the included studies. Most individual studies show a trend favoring rivaroxaban, with a few crossings the line of no effect, but the pooled result favors rivaroxaban for reducing DVT recurrence.

### Recurrence of PE

**Figure 8.**
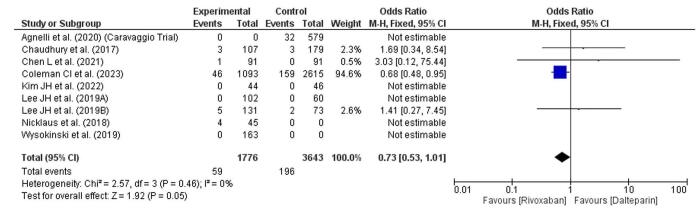
Forest plot showing recurrence of PE.

This forest plot examines the recurrence of PE between rivaroxaban and dalteparin treatments (Fig. 8). The overall OR is 0.73 (95% CI: 0.53–1.01), indicating a 27% lower odds of PE recurrence with rivaroxaban compared to dalteparin, though this result is marginally nonsignificant (*P* = 0.05). The CI narrowly crosses the line of no effect (OR = 1), reflecting the borderline nature of this finding. There is no observed heterogeneity among studies (*I*^2^ = 0%), suggesting consistency across the data sources. Most individual studies indicate a trend favoring rivaroxaban for reducing PE recurrence, although this result should be interpreted cautiously given the nonsignificant *P*-value.

### ROB assessment

**Figure 9.**
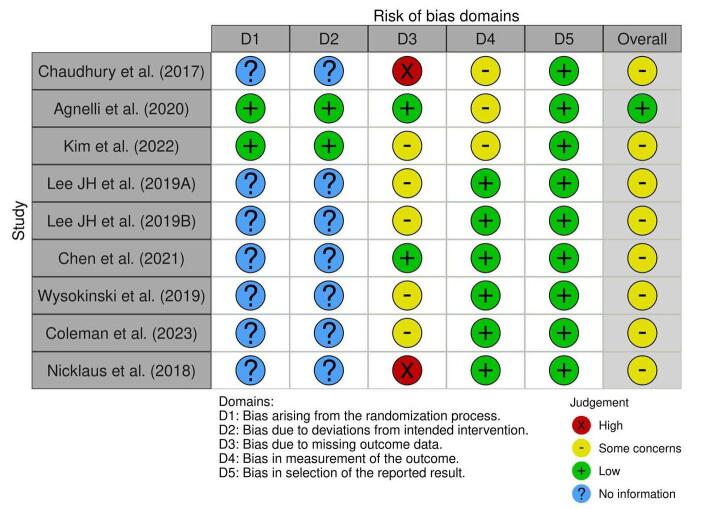
Risk of bias assessment.

**Figure 10.**
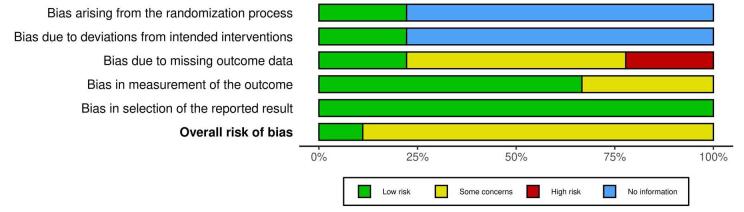
Summary risk of bias assessment.

The ROB assessment across RCTs, observational studies, and cohort studies presented in these images indicate variability in study quality, particularly in specific bias domains. Specifically, many studies exhibit low bias in “Bias due to deviations from intended interventions” (D2), “Bias in measurement of the outcome” (D4), and “Bias in selection of the reported result” (D5), indicating robust adherence to intervention protocols, reliable outcome measurement, and transparency in reporting outcomes. However, “Bias arising from the randomization process” (D1) and “Bias due to missing outcome data” (D3) have notable information gaps, with several studies marked as “No information,” particularly impacting D1, suggesting incomplete data on the randomization quality which might be due to Retrospective study design. Additionally, some studies, like Chaudhury *et al* (2017) and Wysokinski *et al* (2019), show higher concerns or high bias in D3, potentially impacting the integrity of outcome data (Figs. 9 and 10). Overall, there is a moderate concern about bias across studies, especially regarding randomization and missing data, potentially influencing the reliability of aggregate findings.

## Discussion

VTE is a significant complication in patients with cancer, characterized by a markedly increased incidence and recurrence rate compared to the general population. The prevalence of VTE in cancer patients ranges from 3% to 15% per year, depending on the type of malignancy and the treatment regimen.^[25,26]^ This heightened risk is attributed to a cancer-induced hypercoagulable state, which is exacerbated by factors such as chemotherapy, surgery, and immobility.^[27]^ Specifically, the risk of developing VTE is particularly pronounced in the initial months following a cancer diagnosis, with studies indicating that the incidence of VTE is highest during this period.^[28]^ Moreover, cancer patients experience higher rates of recurrent VTE and major bleeding events. The recurrence rate of VTE in this population can reach up to 20% within 12 months after the initial event.^[29]^ This is compounded by the fact that the case fatality rates for recurrent VTE and major bleeding are notably high, particularly within the first three months of anticoagulation therapy. For instance, a meta-analysis indicated that the case fatality rates of recurrent VTE and major bleeding were 3.7% and 6.1%, respectively, during the initial treatment phase.^[30]^ Furthermore, the risk of bleeding complications is significantly elevated in cancer patients, with studies showing that the rates of major bleeding can be as high as 6% in those treated with low molecular weight heparin.^[31]^

The management of VTE in cancer patients is complex due to the dual risks of thrombosis and bleeding. DOACs have emerged as a viable treatment option, demonstrating efficacy in reducing the recurrence of VTE while maintaining a manageable safety profile.^[32]^ Studies have shown that DOACs like rivaroxaban and apixaban are effective in preventing recurrent VTE in cancer patients, with rates of recurrence reported at approximately 4.2%.^[33]^ In terms of major bleeding, the SELECT-D trial indicated that rivaroxaban was associated with a higher incidence of clinically relevant nonmajor bleeding compared to dalteparin (6% vs. 4%). However, the rates of major bleeding were similar between the two treatments, suggesting that while rivaroxaban may lead to more nonmajor bleeding events, it does not significantly increase the risk of major bleeding.^[11,34]^ In a systematic review, major bleeding rates were found to be comparable between rivaroxaban and dalteparin, reinforcing the notion that both anticoagulants have a similar safety profile regarding severe bleeding events.^[34]^ However, in our study, the overall RR is 0.91 (95% CI: 0.57–1.45), indicating no significant difference in major bleeding risk between the two treatments. The pooled effect, also nonsignificant (*P* = 0.69), does not favor either treatment regarding major bleeding risk. Regarding bleeding-related mortality, the data are less definitive. The SELECT-D trial did not report significant differences in mortality related to bleeding between rivaroxaban and dalteparin, although the overall mortality rates in cancer patients with VTE are notably high due to the underlying malignancy.^[11,35]^ This suggests that while bleeding can contribute to mortality, the direct comparison between these two agents does not highlight a significant difference in this regard. In our study, the pooled effect test (*P* = 0.36) is also nonsignificant, suggesting no definitive difference in bleeding-related mortality between the treatments. When considering nonsignificant bleeding events, rivaroxaban has been associated with a higher rate of clinically relevant nonmajor bleeding compared to dalteparin, as previously mentioned.^[11,16]^ This distinction is important for clinicians when weighing the risks and benefits of each anticoagulant, especially in a population that may already be at risk for bleeding due to cancer treatments. In our study, overall OR is 1.18 (95% CI: 0.88–1.57), indicating a slightly higher, yet statistically nonsignificant, likelihood of nonsignificant bleeding events in the Rivaroxaban group compared to dalteparin. In terms of recurrence rates for DVT and PE, rivaroxaban has shown a lower recurrence rate compared to dalteparin. In the SELECT-D trial, the recurrence of VTE at 6 months was significantly lower in the rivaroxaban group (4%) compared to the dalteparin group (11%).^[11,35]^ This finding is consistent with other studies that have reported rivaroxaban’s efficacy in reducing VTE recurrence in cancer patients.^[16]^ In our study, most individual studies show a trend favoring rivaroxaban, with a few crossing the line of no effect, but the pooled result favors rivaroxaban for reducing DVT recurrence.

Current data are limited by the relatively small sample sizes in individual studies, which impacts the precision and generalizability of findings, especially regarding bleeding-related mortality and major bleeding risks. Future research should focus on conducting large-scale, cancer-specific RCTs to assess the comparative efficacy and safety of rivaroxaban and dalteparin in different cancer types and stages, along with observational studies capturing real-world patient outcomes, including quality of life and adherence. Studies on optimal dosage, treatment duration, and long-term impacts on survival and quality of life are essential, as well as cost-effectiveness analyses to evaluate healthcare resource utilization. Research should also explore the potential for combination or sequential therapy approaches to enhance efficacy while minimizing adverse effects, with the goal of developing standardized, consensus-based guidelines for cancer-associated VTE management that support personalized, evidence-based care.

## Conclusion

The study concludes that rivaroxaban is a viable alternative to dalteparin for managing cancer-associated VTE, offering comparable safety in terms of major bleeding risk and potentially superior efficacy in reducing DVT recurrence. Despite a slightly higher but nonsignificant rate of minor bleeding events with rivaroxaban, its oral administration and patient adherence benefits make it a favorable option. However, further research is needed to establish long-term outcomes and refine anticoagulant selection guidelines for cancer-associated VTE management.

## Data Availability

The datasets used and/or analyzed during the current study are available from the corresponding author upon reasonable request.

## Research registration unique identifying number (UIN)

Research registry 10806.
